# Is the methanogenic community reflecting the methane emissions of river sediments?—comparison of two study sites

**DOI:** 10.1002/mbo3.454

**Published:** 2017-03-16

**Authors:** Prem Prashant Chaudhary, Martin Rulík, Martin Blaser

**Affiliations:** ^1^ Department of Internal Medicine University of Michigan Ann Arbor MI USA; ^2^ Department of Ecology and Environmental Sciences Faculty of Science Laboratory of Aquatic Microbial Ecology Palacky University Olomouc Czech Republic; ^3^ Department of Biogeochemistry Max Planck Institute for Terrestrial Microbiology Marburg Germany

**Keywords:** depth profile, mcrA, methanogen, qPCR, T‐RFLP

## Abstract

Studies on methanogenesis from freshwater sediments have so far primarily focused on lake sediments. To expand our knowledge on the community composition of methanogenic archaea in river sediments, we studied the abundance and diversity of methanogenic archaea at two localities along a vertical profile (top 50 cm) obtained from sediment samples from Sitka stream (the Czech Republic). In this study, we compare two sites which previously have been shown to have a 10‐fold different methane emission. Archaeal and methanogen abundance were analyzed by real‐time PCR and T‐RFLP. Our results show that the absolute numbers for the methanogenic community (qPCR) are relatively stable along a vertical profile as well as for both study sites. This was also true for the archaeal community and for the three major methanogenic orders in our samples (Methanosarcinales, Methanomicrobiales, and Methanobacteriales). However, the underlying community structure (T‐RFLP) reveals different community compositions of the methanogens for both locations as well as for different depth layers and over different sampling times. In general, our data confirm that Methanosarcinales together with Methanomicrobiales are the two dominant methanogenic orders in river sediments, while members of Methanobacteriales contribute a smaller community and Methanocellales are only rarely present in this sediment. Our results show that the previously observed 10‐fold difference in methane emission of the two sites could not be explained by molecular methods alone.

## 1 Introduction

River sediments are an example of a unique type of ecosystem which is structured longitudinally as well as vertically and is affected by the fluctuating availability of decayed organic matter coming mostly from the surrounding terrestrial environment. Depending on the local conditions, the decaying organic matter can either be oxidized to CO_2_ if oxygen is present or can be anaerobically fermented to CO_2_ and methane if other electron acceptors like nitrate, iron, and manganese are depleted. Current data suggest that rivers contribute about 3% of the total release of methane into the atmosphere (Saarnio, Winiwarter, & Leitao, 2009) or 15%–40% of the efflux of wetland and lakes (Stanley et al., 2016). The majority of this methane is produced in anoxic environments by methanogenic archaea (Bastviken, Cole, Pace, & Tranvik, 2004; Ciais et al., 2014; Wuebbles & Hayhoe, 2002). Generally, the mineralization of the organic matter under anaerobic conditions is carried out by several microbial organisms: Initially, the organic matter is depolymerized and then the monomers are fermented to CO_2_ and short‐chain fatty acids alcohols and other substances, which in turn can be further degraded by syntrophic organisms to finally H_2_, CO_2_ and acetate (Schink, 1997). In the absence of other electron acceptors like nitrate, iron, manganese, etc., the terminal step of the anaerobic organic matter mineralization results in the release of methane and CO_2_ (Schink, 1997; Zeikus, 1983).

Methanogens are considered to be of prime importance because they are responsible for the final step of mineralization of organic carbon to methane (CH_4_) (Capone & Kiene, 1988; Delong, 1992). Methane is one of the most potent greenhouse gases with a global warming potential 25 times higher than carbon dioxide. A significant contribution to the annual atmospheric methane flux (40%–50%) comes from freshwater sediments like lakes, wetlands, and rice paddy fields (Cicerone & Oremland, 1988; Conrad, 2009; Rulik et al., 2013). As the sediment depth increases, there is also a shift in the physical and chemical conditions, such as redox potential and dissolved oxygen, an increase in temperature and nutrient gradients, which constitutively provides a unique environment for the growth of metabolically diverse microorganisms (Chunleuchanon, Sooksawang, Teaumroong, & Boonkerd, 2003; Newberry et al., 2004; Orphan et al., 2008).

In a previous study, we already evaluated the methane emissions as well as the methanogenic potential of several sites of River Sitka (Rulik et al., 2013). In the present study, we focused on the methanogenic community composition of river sediment samples and compare the community composition of a low‐emitting site (Location I: 2.39 mg CH_4_ m^−2^ water day^−1^) with that of a high‐emitting site (Location IV: 32.1 mg CH_4_ m^−2^ water day^−1^) (Rulik et al., 2013).

Currently, there are seven orders of methanogenic archaea described in literature (Borrel et al., 2013, 2014; Lang et al., 2015). However, our previous study conducted on the Sitka stream (Location IV) revealed only three major methanogenic groups using molecular techniques (denaturing gradient gel electrophoresis and cloning): Methanosarcinales, Methanomicrobiales, and Methanobacteriales (Brablcova, Buriankova, Badurova, Chaudhary, & Rulik, 2014; Buriankova et al., 2013; Chaudhary et al., 2014). Hence, we focused our attempts to verify these results with molecular fingerprinting and qPCR to cover these three groups; in addition, we want to expand our knowledge by comparing two different sites and two sampling occasions..

In the Sitka stream, previous studies showed that methanogenic archaea are almost ubiquitous along the longitudinal profile of the stream (Brablcova et al., 2014; Buriankova et al., 2012) and their density tends to be stable with increasing sediment depth (Location IV) (Buriankova et al., 2012). However, quantification of total methanogens was made using fluorescence in‐situ hybridization (FISH) (Buriankova et al., 2012), which is suitable for aqueous systems but may lack precision in sediment samples due to high background fluorescence.

The present study aimed to analyze the vertical distribution of methanogens in the top 50 cm of river Sitka sediment cores from one high and one lower methane‐producing localities, and to quantify the methanogenic communities using a combination of terminal restriction fragment length polymorphism (T‐RFLP) and qPCR. We expected that especially the quantification with qPCR would help not only for total archaea but also for the three dominant methanogenic orders to increase our understanding on the different methane emissions of the two sites. The group specific qPCR has so far not been applied to many environmental systems. Since the *mcrA* primers are highly degenerated to cover a broad community, we hoped to improve our understanding of the system using group specific qPCR. Likewise, our new dataset provided us to contrast our T‐RFLP results with previous work on Location IV (Mach, Blaser, Claus, Chaudhary, & Rulik, 2015) and demonstrate the development of the methanogenic community over one and a half years.

## 2 Material and Methods

### 2.1 Ethics statement

For the collection of sediment samples from the specific sites, no specific permits were required. The locations were not privately owned, nor were they in restricted or protected areas. Moreover, no activities involving endangered or protected species were untaken during the collection of samples.

### 2.2 Study site

Sitka stream is considered to be an undisturbed, 35 km long, lowland, third‐order stream originating in the Hrubý Jeseník Mountains, 650 m above sea level. Of the two localities studied, one (Location I) was situated in an upper forested area, whereas the second location (Location IV) was situated in agricultural landscape (further description of the sampling sites has been provided earlier (Hlavacova, Rulik, & Cap, 2005; Buriankova et al., 2013; Rulik et al., 2013; Brablcova et al., 2014). These two sites were selected on the basis of the different amount of methane production and methanogenic potential on the basis of earlier studies (Buriankova et al., 2012, 2013). Location IV was studied previously in more detail because of maximum methane production and methanogenic potential (Buriankova et al., 2013; Mach et al., 2015). Sediment sampling for studying the vertical distribution of methanogens was performed in July 2013. Three sediment cores (50 cm deep) were taken randomly at each Location I and Location IV, along Sitka stream flowing through Olomouc province in the Czech Republic. The focus of this study was to compare depth profiles of both locations using community profiling (T‐RFLP) as well as quantification of the methanogenic community not only using the commonly used *mcrA* marker gene but also to use group specific primers to quantify the three dominant methanogenic orders.

### 2.3 Collection and processing of sediment sample

Hyporheic sediment samples were collected using the liquid N_2_ freeze‐core method (Bretschko & Klemens, 1986). A total of three cores were gathered and taken for subsequent analyses. After sampling, five layers (i.e., 0–10 cm, 10–20 cm, 20–30 cm, 30–40 cm, and 40–50 cm) were immediately separated for subsequent molecular analysis and stored at low temperature during transport to the laboratory. Samples were then thawed and wet sediment from each layer was sieved and only particles <1 mm were considered for DNA isolation since most of the microorganisms would be attached to them (Leichtfried, 1988; Ramakrishnan, Lueders, Conrad, & Friedrich, 2000). A total of 15 subsamples (three from each depth) were used for DNA extraction. Dry weight of the samples was determined by drying 1 g of the samples at 60°C over night.

### 2.4 DNA extraction and terminal restriction fragment length polymorphism (T‐RFLP) analysis

For genomic DNA extraction, 1 g wet weight of sediment sample was processed using the PowerSoil DNA Isolation Kit (MO‐BIO, USA), according to the manufacturer's instructions. Extracted DNA was checked for quality and concentration using a Nanodrop spectrophotometer (Nano‐Drop Technologies, Wilmington). Terminal restriction fragment length polymorphism (T‐RFLP) analysis of the methanogenic *mcrA* genes was carried out as described previously (Lueders & Friedrich, 2003), using the primer pairs MCRf and MCRr, with the forward primer labeled with FAM (Table 1). The PCR products were purified using the QIAquick PCR purification kit (Qiagen, Hilden, Germany) according to the manufacturer's instructions. Aliquots of the purified amplicons (200 ng) were digested with *Sau*96I (Fermentas). After the digestion, the DNA samples were precipitated in 200 μl of 75% isopropanol for 30 min at room temperature, followed by centrifugation at 14,000*g* for 30 min at 4°C. The DNA pellets were washed with 70% ethanol, air‐dried, and resuspended in 20 μl of purified water. The fluorescently labeled T‐RF were size‐separated on the automatic sequencer ABI 3100 Avant Genetic Analyzer (Applied Biosystems) equipped with POP6 polymer‐filled capillary under denaturing condition. The T‐RFLP electropherograms were analyzed by peak area integration of the T‐RF using the GeneScan analyzing software (Applied Biosystems). The lengths of the T‐RF were determined by comparison to an internal standard (GeneScan‐1000‐ROX size standard; Applied Biosystems). The relative abundance of a single T‐RFLP was represented by the percentage fluorescence intensity calculated relative to the total fluorescence intensity of all well‐resolved peaks with area over 1,000 or >2% of the maximum peak of an electropherogram. The possible phylogenetic affiliations were determined by comparison of the T‐RFLP length of clones of the sediment samples (Mach et al., 2015) to the theoretical T‐RFLP lengths generated from the sequences deposited in GenBank database using Ribosomal Database Project T‐RFLP online analysis.

**Table 1:** Characteristics of primer sets used in quantitative PCR and T‐RFLP

Name	Target group	Sequence (5′–3′)	Annealing temperature (°C)	Amplicon size (bp)	Reference
PARCH340‐F PARCH519‐R	Archaea (qPCR)	CCC TAC GGG GYG CAS CAG TTA CCG CGG CKG CTG	58.3	152	(Ovreas et al., 1997)
MCRA‐F MCRAR‐ R	Methanogens (qPCR)	GGT GGT GTM GGD TTC ACM CAR TA TTC ATT GCR TAG TTW GGR TAG TT	55	488	(Luton et al., 2002)
MBT857‐F MBT1196‐R	Methanobacteriales (qPCR)	CGW AGG GAA GCT GTT AAG T TAC CGT CGT CCA CTC CTT	53.4	342	(Yu et al., 2005)
MMB282‐F MMB832‐R	Methanomicrobiales (qPCR)	ATC GRT ACG GGT TGT GGG CAC CTA ACG CRC ATH GTT TAC	50.7	506	(Yu et al., 2005)
MSL812‐F MSL1159‐R	Methanosarcinales (qPCR)	GTA AAC GAT RYT CGC TAG GT GGT CCC CAC AGW GTA CC	52.7	354	(Yu et al., 2005)
mcrA‐F(FAM Labelled) mcrA‐R	Methanogens (T‐RFLP)	TAY GAY CAR ATH TGG YT ACR TTC ATN GCR TAR TT	50	516	(Springer, Sachs, Woese, & Boone, 1995)

### 2.5 qPCR analysis

In order to quantify the microbial community, we used a set of different primers targeting the total archaea (16S *rRNA* genes), methanogenic archaea (*mcrA* gene), and three major methanogenic orders Methanobacteriales (MBT‐set), Methanomicrobiales (MMB‐set), or Methanosarcinales (MSL‐set) (Luton, Wayne, Sharp, & Riley, 2002; Ovreas, Forney, Daae, & Torsvik, 1997; Yu, Lee, Kim, & Hwang, 2005) (Table 1). qPCR was performed using the BioRad CFX Connect^™^ qPCR detection system (BioRad, USA). The 25 μl real‐time PCR mixture was prepared using the Brilliant II SYBR master mix (Agilent Technologies, USA) 12.5 μl of 2× reaction solution, 0.25 μl of each primer (final concentration 0.25 μmol/L), 5 μl of template DNA, and 7 μl of PCR‐grade water. The two‐step amplification protocol was as follows: initial denaturation for 5 min at 94°C followed by 45 cycles of 30 s at 94°C and combined annealing and extension for 30 s at XºC (X values are given in Table 1). The fluorescent signal was measured at the end of each annealing/extension step. DNA samples were analyzed in triplicate at each point.

In order to generate standard curves, target genes were amplified with PCR. The PCR products were cloned into the pGEM‐T Easy vector (Promega, Madison, WI). The plasmids were extracted, serially diluted, and used as templates in qPCR for generating standard curves.

## 3 Results

### 3.1 Quantification (qPCR) of archaeal, *mcrA* gene copies and three orders of methanogens

**Figure 1:**
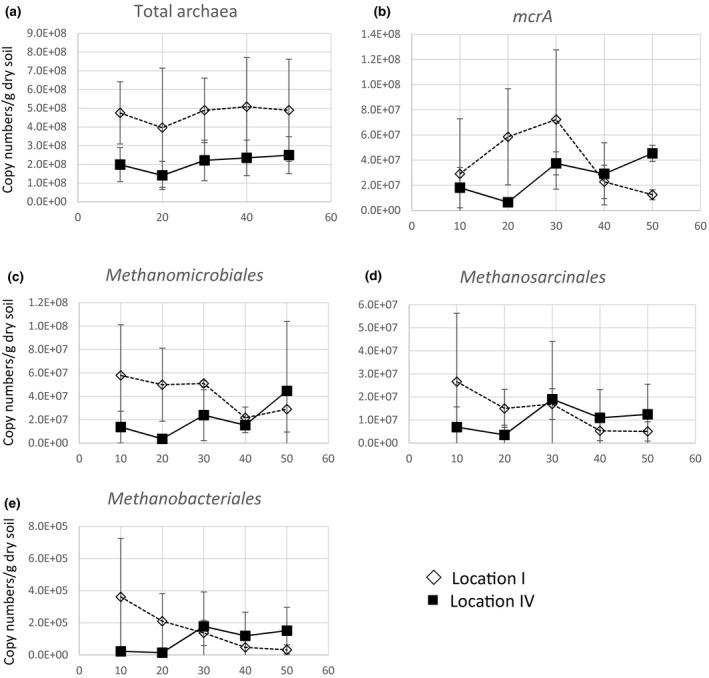
qPCR results given as copy numbers per gram dry weight of a) total archaea (*16S RNA*), b) total methanogens (*mcrA*), c) Methanomicrobiales, d) Methanosarcinales, e) Methanobacteriales. For different depth (10 = 0–10 cm, 20 = 10–20 cm, 30 = 20–30 cm, 40 = 30–40 cm, 50 = 40–50 cm) for Location I and Location IV of Sitka river sediments. Comparison of different genes for the two locations can be found in the supplementary as Figure S1

The measurements were made for all five depths of the two localities I and IV (i.e., 0–10, 10–20, 20–30, 30–40, and 40–50 cm of depth) (Figure 1); an overview of the qPCR results for the individual locations can be found in the supplementary as Figure S1. Archaeal densities were found to be in the range of 10^8^ copies/g dry weight with a slight increase in density as the depth increases (Figure 1a). The copy numbers of the *mcrA* gene characteristic for the methanogens, remained stable at around 10^7^ copies/g dry weight at all depths for Location I and IV (Figure 1b). A slight increase in the copy numbers at 20 and 30 cm depths can be seen from the samples at locality I (Figure 1b), followed by a decrease at 40 and 50 cm of depth. However, for Location IV, *mcrA* gene numbers were slightly greater at 50 cm depth as compared to 40 cm depth.

The highest copy numbers for the analyzed methanogenic orders belonged to the order Methanomicrobiales (Figure 1c). Here, from 3.6*10^6^ to 5.8*10^7^ copies/g dry weight could be reported. While the average copy numbers slightly decreased with depth in Location I; they slightly increased in Location IV. Gene copy numbers of methanogens belonging to the order Methanosarcinales were in a similar range covering from 3.6*10^6^ to 2.7*10^7^ copies/g dry weight (Figure 1d). In Location I, again a slight decrease with depth could be observed, while in Location IV, a maximum at 20–30 cm was observed. Methanogens belonging to the order Methanobacteriales were found with roughly two orders of magnitude lower copy numbers ranging from 1.4*10^4^ to 3.6*10^5^ copies/g dry weight (Figure 1e). Again, a decrease was observed over the different depth at Location I while a slight increase was reported for Location IV. Irrespective of the tested methanogenic order all three primer sets revealed a decrease over depth in methanogenic copy numbers per gram dry weight for Location I (Figure S1) while all three sets gave consistently low copy numbers for the 10–20 cm depth samples at Location IV.

### 3.2 Terminal restriction length polymorphism of *mcrA* genes

**Figure 2:**
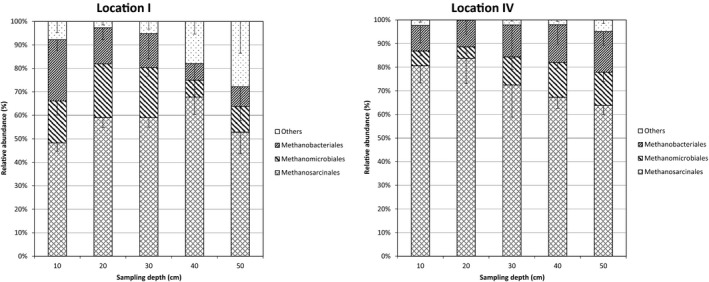
Community profile using T‐RFLP of *mcrA* for both locations. Results are given on the order level, details for individual TRF's can be found in the supplementary as Figure S2

The methanogenic community composition was determined by analysis of the terminal restriction fragment length polymorphism (T‐RFLP) of the *mcrA* gene in both localities (I and IV) at the five different depths (Figure 2). The T‐RFLP‐profiles show 8–13 different TRF's (Figure S2). The relative contribution of the order Methanosarcinales to total methanogenic TRF's was almost always dominant contributing 48%–84% of the total TRF's. While the relative contribution of Methanosarcinales decreased with sediment depths at Location IV, it had a maximum at 40 cm for the samples taken at Location I. A closer look on the six TRF's assigned to the Methanosarcinales (252–3 bp, 390–1 bp, 415–7 bp, 423–427 bp, and 491–2 bp, 504–6 bp) revealed that the top sediments at Location IV was dominated by a single TRF (491–2 bp) while Location I showed a different dominating TRF (504–6 bp) for the 30–40 cm depth layer (Figure S2).

The relative contribution of methanogens belonging to the order Methanobacteriales increased with sediment depth reaching from 11% to 17% in Location IV; at Location I, their values decreased from 26% to 8% over the sediment depth. Only, one TRF (400–3) could be assigned to Methanobacteriales.

The relative abundance of the third methanogenic order Methanomicrobiales ranged from 5% to 23% and did not show a clear trend over the different depth of the sediment profile. Four TRF's (324–5 bp, 405–406 bp, 410 bp and 472–4 bp) could be attributed to this order.

While most of the TRF's found in Location IV could be attributed to the three dominant methanogenic orders, up to 28% of the TRF's in Location I (mainly TRF 366 bp) could not be assigned to any known methanogen.

**Figure 3:**
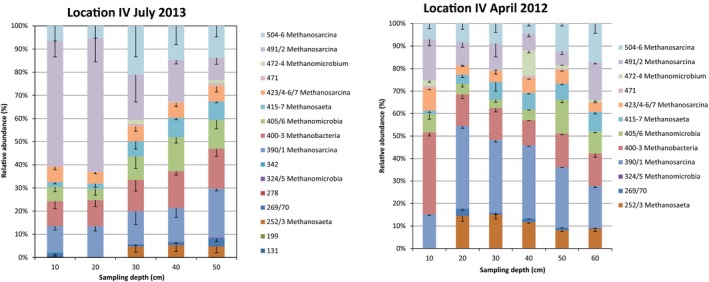
Comparison of the community profile (T‐RFLP of *mcrA* gen) for the depth profile of two sediment cores from different sampling time points of the high methane‐emitting site (Location IV). The samples from April 2012 have been previously evaluated in a different context (Mach et al., 2015)

Rivers are very dynamic systems; hence, we wanted to compare the temporal changes of the methanogenic community at the high methane‐emitting site. A comparison of cores taken at Location IV in April 2012 and July 2013 reveals that the community profiles are rather stable over the different depth layers (Figure 3). However, the relative contribution of individual TRF's is quite different over time. For example, the 491–2 bp TRF which contributes 54%–58% to the community of the top 20 cm in July 2013 represents only 10%–18% in the earlier samples. Likewise, several minor TRF's which have been reported for the top layer of the samples taken in July 2013 (TRF 131, 199, 278, 342) have not been found in the samples taken in April 2012.

## 4 Discussion

Although methanogenesis is one of the main processes responsible for terminal anaerobic organic matter mineralization in the river hyporheic sediments (Hlavacova et al., 2005), very little is known about the methanogens involved in this process. One would expect that the diversity of the methanogenic community should to some extent reflect the level of methanogenic production. However, microbial diversity and how it correlates with the function in the sediments are not trivial. Moreover, the diversity and composition of the methanogenic community might change along the longitudinal profile, as well as along the vertical profile of the stream (Brablcova et al., 2014).

### 4.1 Contribution of methanogenic archaea to total microorganisms/archaea in freshwater sediments

In lake sediments, archaea account from less than 1% (Schwarz, Eckert, & Conrad, 2007) to 96.9% (Ye et al., 2009) of the prokaryotic community when comparing qPCR results of the archaeal 16S rRNA gene to the bacterial counterpart. Our previous data from a vertical profile of the Sitka sediments indicated a relative contribution of 13.8%–14.7% of archaea to the overall microbial community (Buriankova et al., 2012).

While the archaeal abundance has been reported to either decrease (Chan et al., 2005) or increase with depth of sediments (Kotsyurbenko et al., 2004), it was rather constant in our study. The methanogenic (*mcrA* copy numbers) contribution to the archaeal community was roughly 10% (ranging from 2.5% to 14.8% in Location I, and 4.6 to 18.2% in Location IV).

### 4.2 Methanogenic community in river sediments analyzed by different molecular techniques

The methanogenic community based on T‐RFLP of *mcrA* has so far primarily been described for rice field soils (Chin, Lueders, Friedrich, Klose, & Conrad, 2004; Conrad, Klose, Noll, Kemnitz, & Bodelier, 2008; Kemnitz, Chin, Bodelier, & Conrad, 2004; Lueders, Chin, Conrad, & Friedrich, 2001; Ramakrishnan, Lueders, Dunfield, Conrad, & Friedrich, 2001). While our previous studies of river Sitka sediments using T‐RFLP (Mach et al., 2015) already show that the community pattern changes over the depth profile, we wanted to confirm these results for two locations and further support them using order specific qPCR. However, the results can not directly be compared since T‐RFLP is based on the highly degenerated *mcrA* primers and only gives relative abundances, while the order specific primers for qPCR gives absolute numbers for the respective methanogenic order according to the standards used. In addition, the primers used for T‐RFLP target a different region of the *mcrA* gene than the ones used for qPCR of *mcrA*. Both primer sets are wobbled to allow a broad coverage. The group specific primers are much more precise, and hence, the sum of the copy numbers obtained for the three groups is up to 1.6 times higher than the results obtained by the general *mcrA* primer set making a relative quantification of the qPCR results difficult. While both methods are consistently showing a dominance of Methanosarcinales, Methanomicrobiales likewise have high copy numbers and contribute between 5% and 23% of the TRF's (and from 15% to 50% of the qPCR). Methanobacteriales have two orders of magnitude lower copy numbers (Figure S1) and contribute only one TRF. However, this TRF (400–3 bp) accounts for up to 26% of the methanogenic community shown for the top sediment of Location I (Figure S2).

Our previous study conducted on the Sitka stream also revealed phylotypes from the orders Methanosarcinales, Methanomicrobiales, and Methanobacteriales (Brablcova et al., 2014; Buriankova et al., 2013; Chaudhary et al., 2014). A community profiling using denaturing gradient gel electrophoresis DGGE presented by Brablcova et al., (2014) showed nine bands for Methanosarcinales, one band for Methanomicrobiales, and one band for Methanocellaceae. It is interesting to note the one clone obtained for Methanocellaceae (Brablcova et al., 2014) originates from Location I, and only for this location, we could assign one TRF (238 bp) to Methanocellaceae for the 40–50 cm depth confirming the presence of this microbial order in the sediments of Location I. A microscopic study using FISH of Methanosarcinaceae, Methanosaetaceae as well as Methanobacteriaceae, not only revealed the presence of these three groups with each contributing roughly 10% to the total cell counts (DAPI counts) (Rulik et al., 2013), but also showed that the vertical distribution is quite stable.

The currently available two clone libraries for the Sitka river sediments (Buriankova et al., 2013; Mach et al., 2015) show both a dominance of Methanosarcinales (47%–56% of the clones), the second equally important group was Methanomicrobiales covering 40%–42% of the clones; a less frequently found order was Methanobacteriales with 4%–10% of the clones. Together these data demonstrate that Methanosarcinales are the dominant order in the Sitka River sediments followed by Methanomicrobiales and Methanobacteriales. A smaller clone library (Brablcova et al., 2014) confirmed the dominant contribution of *Methanosarcinales* (6 out of 11 clones).

Likewise, in other environmental samples, Methanosarcinales and Methanomicrobiales have been described as dominant methanogenic members using various archaea/methanogen‐specific primers, e.g., from river freshwater and estuarine sediment (Brablcova et al., 2014; Buriankova et al., 2013; Munson, Nedwell, & Embley, 1997; Purdy, Munson, Nedwell, & Embley, 2002), as well as from peat bog sites (Galand, Fritze, Conrad, & Yrjala, 2005), freshwater lake sediments (Falz et al., 1999; Koizumi, Takii, & Fukui, 2004), Florida Everglades wetland soils (Castro, Ogram, & Reddy, 2004), hydrocarbon‐contaminated aquifer (Kleikemper et al., 2005), and deep‐sea hydrothermal sediments (Dhillon et al., 2005).

In general, our results are in good agreement with reported methanogenic community profiles of other freshwater habitats (e.g., lakes) which usually are also dominated by Methanomicrobiales and Methanosarcinales (Banning et al., 2005; Barreto, Conrad, Klose, Claus, & Enrich‐Prast, 2014; Castro, Newman, Reddy, & Ogram, 2005; Conrad et al., 2014). In contrast, the T‐RFLP profiles of rice field soil are more diverse and contain additional methanogenic orders (Chin et al., 2004; Conrad et al., 2008; Kemnitz et al., 2004; Lueders et al., 2001; Ramakrishnan et al., 2001).

### 4.3 Comparison of the vertical distribution and composition of the methanogenic community

The different depth profiles show that the major methanogenic orders are relatively stable over the analyzed top 50 cm of the sediment (Figure 2). This is in agreement with the previously published T‐RFLP profile for Location IV (sampled at a different year) (Mach et al., 2015). Only a finer resolution of the different TRF's shows that the members of the different orders vary for different depth as well as for the two sampled locations (Figure S2). A recent study on the methanogenic community of the Yangtze River estuary using 454 pyrosequencing also shows that in this river sediment Methanosarcinales as well as Methanomicrobiales are the dominant members of the methanogenic community (Zeleke et al., 2013). In this study, they also analyzed the *mcrA* copy numbers/g dry weight and confirm the overall picture of relatively stable 10^7^–10^8^ copies for the top 50 cm. Only at deeper sediment depth they found an increase in *mcrA* copies (Zeleke et al., 2013), which is in agreement with our results. In addition, we could show that even for the three tested methanogenic orders we generally find quite stable copy numbers for both locations as well as over the different depth (Figure 1).

If we compare both locations, we see that the overall *mcrA* copy numbers (as well as the group specific copy numbers) are relatively stable along the depth profiles. Astonishingly, the lower methane‐emitting site (Location I) has on average higher cell counts for all tested methanogenic groups when compared to the higher methane‐emitting site (Location IV). This suggests that the activity of the methanogenic community is rather controlled by other factors (e.g., substrate supply) than by size of the community.

The detailed methanogenic community profile (Figure S2) is different for both locations and changes over the depth profile of the sediment cores. While a core set of seven TRF's was reported for both locations, individual TRF's were only present in one of the two sampling sites (e.g., TRF 366 bp (others) Location I, 410 bp (Methanomicrobia*)* Location I, 491/2 bp (*Methanosarcina*) Location IV) (Figure S2).

Likewise, we could report a change in the community profile comparing samples from April 2012 and July 2013. Currently, it cannot be excluded that these differences are due to seasonal variations.

Looking at the relative stable copy numbers and the methanogenic community profile, one may assume that the different depth as well as the different locations will show similar methanogenic potentials. Our previous studies, however, show that the methanogenic potential for Location IV showed two distinct activity peaks (for the top sediment as well as the 40–50 cm depth) (Mach et al., 2015); likewise, the methane emissions for both locations is quite distinct providing evidence that Location IV is a 10 times stronger methane‐emitting site (Rulik et al., 2013). This suggest that the methanogenic potential is not only limited by the presence of the different methanogens but also more likely regulated by environmental factors (e.g., substrate supply) as well as the activity of certain members of the methanogenic community. Hence, fine resolved studies like the presented T‐RFLP profiles or next generation sequencing data are needed to fully resolve the complex processes involved in the methane release from river sediments.

## 5 Conclusions

Data obtained in this study validated our previous measurements for Location IV on the composition and diversity of the methanogenic archaea within the hyporheic sediments of the Sitka stream and contrasted these results to a lower methane‐emitting site (Location I). Generally, this study confirms that methanogens are ubiquitous members of the microbial community within river hyporheic sediments. The richness of the methanogenic community is less diverse in river sediments compared to those from wetlands or rice paddies.

Our results show that the methanogenic community in methane‐emitting river sediments is relatively stable in absolute numbers along a vertical profile and for both study sites (irrespective of the reported methane emissions) not only on the level of total archaea and total methanogens but also likewise on the level of the three dominant methanogenic orders. Especially, the quantification of different methanogenic orders has so far not been applied to river sediment samples and provides additional evidence for the quantification of the individual methanogens. However, the underlying community structure reveals different community compositions of the methanogens for both locations as well as for different depth layers and different sampling times. In general, our data confirm that Methanosarcinales together with Methanomicrobiales are the two dominant methanogenic orders in river sediments, while members of Methanobacteriales contribute a smaller community and Methanocellales are only rarely present in this sediment.

## Conflict of interest

The authors declare that there is no conflict of interest regarding the publication of this paper.
