# Supplementary material for: Is the methanogenic community reflecting the methane emissions of river sediments?—comparison of two study sites
Source: Microbiologyopen. 2017 Mar 16;6(4):e00454. doi: 10.1002/mbo3.454 (PMC5552910; doi:10.1002/mbo3.454)
Supplement: Supplementary file 1 [file MBO3-6-na-s001.doc]

**Is the methanogenic community reflecting the methane emissions of river sediments? – Comparison of two study sites**

**Supplementary**

Prem Prashant Chaudhary^1^, Martin Rulík^2^, and Martin Blaser^3^

^1^Department of Internal Medicine, University of Michigan, Ann Arbor MI, USA ²Department of Ecology and Environmental Sciences, Faculty of Science, Laboratory of Aquatic Microbial Ecology, Palacky University, Šlechtitelů 11, 783 71 Olomouc, Czech Republic; ^3^Department of Biogeochemistry, Max Planck Institute for Terrestrial Microbiology, Marburg, Germany

*Corresponding author:

Martin Blaser

Max Planck Institute for Terrestrial Microbiology

Karl-von-Frisch-Str. 10

35043 Marburg, Gemany

Tel: +49-6421-178 820

Fax: +49-6421-178 999

Email: blaserm@mpi-marburg.mpg.de

**
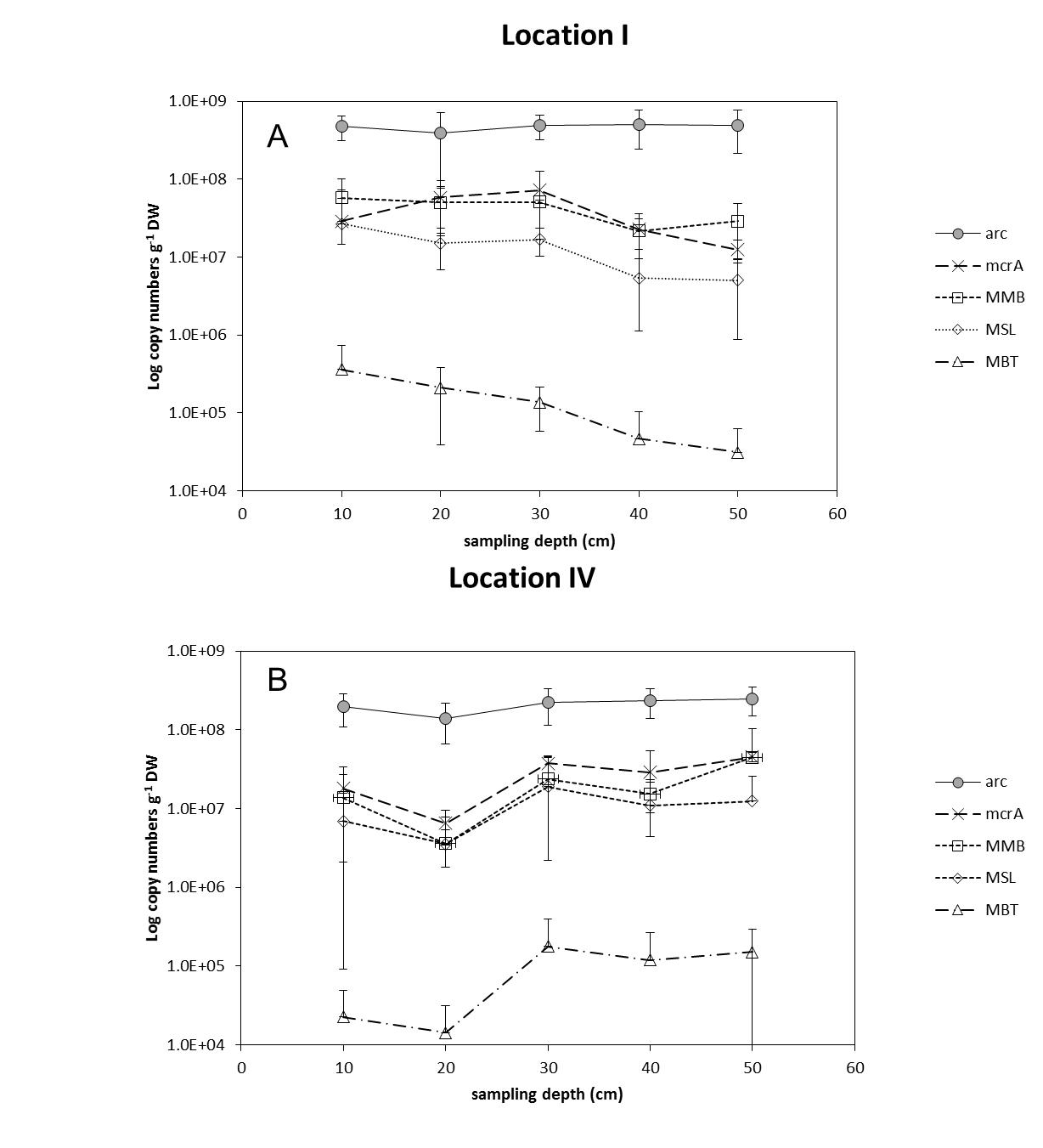
**

**Figure S1.** Comparative qPCR results of the two locations showing the copy numbers of archaea (16S RNA), total methanogens (*mcrA*), *Methanomicrobiales* (MMB), *Methanosarcinales* (MSL), *Methanobacteriales* (MBT) for different sediment depth (10 = 0-10 cm; 20 = 10-20 cm; 30 = 20-30 cm; 40 = 30-40 cm; 50 = 40-50 cm). Results are given on a logarithmic scale as average ± SD (n=3).


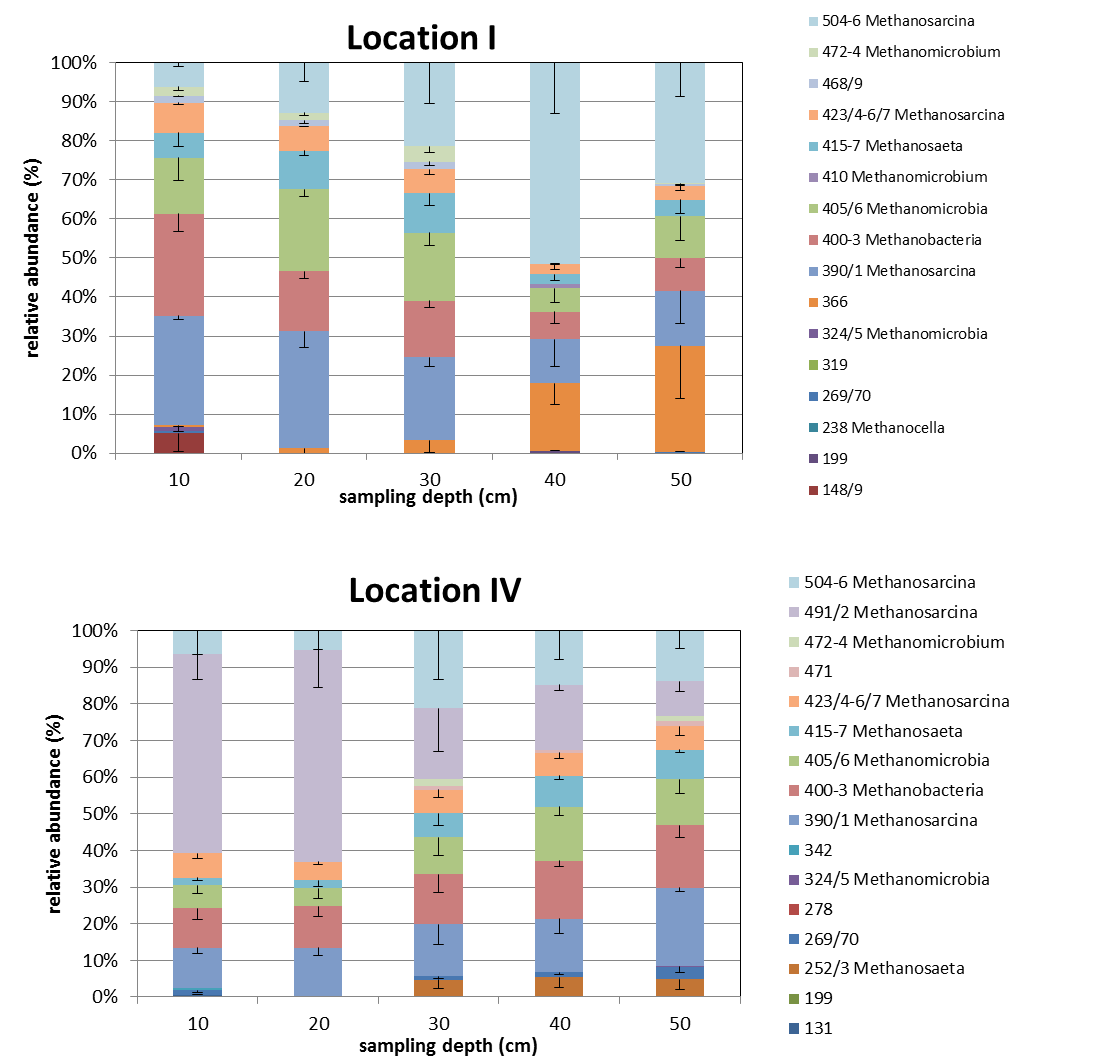


**Figure S2** Detailed relative abundance of methanogenic community based on TRFLP of the *mcrA* gene. Data is shown as average ± SD (n=3) for different depth (10 = 0-10 cm; 20 = 10-20 cm; 30 = 20-30 cm; 40 = 30-40 cm; 50 = 40-50 cm) of both sampling locations. The TRF’s were assigned to different methanogens according to a published clone library of river sediments ; the 415-417bp fragment has been assigned as *Methanosaeta* using .

References

Conrad R., Klose M., Noll M., Kemnitz D. & Bodelier P.L.E. (2008) Soil type links microbial colonization of rice roots to methane emission. *Global Change Biology,* **14,** 657-669.

Mach V., Blaser M.B., Claus P., Chaudhary P.P. & Rulik M. (2015) Methane production potentials, pathways, and communities of methanogens in vertical sediment profiles of river Sitka. *Front Microbiol,* **6,** 506.

Ramakrishnan B., Lueders T., Dunfield P., Conrad R. & Friedrich M.W. (2001) Archaeal community structures in rice soils from different geographical regions before and after initiation of methane production. **37,** - 186.
